# From Cold Fingers to Stroke: A Rare Pediatric Case of Left Atrial Myxoma Revealed by Raynaud’s Phenomenon

**DOI:** 10.7759/cureus.93990

**Published:** 2025-10-06

**Authors:** Yasmine Elhajji, Fatima-zahrae Jamali, Mohamed Rida Ajaja, Loubna Chtouki, Younes Cheikhaoui

**Affiliations:** 1 Cardiology, Cheikh Zaid Hospital, Rabat, MAR; 2 Pediatric Cardiac Surgery, Cheikh Zaid Hospital, Rabat, MAR; 3 Pediatrics, Mohammed V University, Rabat, MAR

**Keywords:** cardio thoracic surgery, large atrial myxoma, myxoma surgical resection, pediatric stroke, raynaud’s phenomenon

## Abstract

Primary cardiac tumors are extremely rare in children, with atrial myxomas representing only a small subset. We report the case of a seven-year-old boy initially followed for Raynaud’s phenomenon, who later presented with acute ischemic stroke. Neurological examination and MRI confirmed left hemispheric infarction. Transthoracic echocardiography revealed a large, mobile mass occupying the left atrium and prolapsing into the left ventricle, highly suggestive of myxoma. Emergency surgical resection was performed, achieving complete excision of the tumor with uneventful recovery. Histopathological analysis confirmed a left atrial myxoma. This case illustrates the diagnostic challenge of pediatric myxomas, which can present with nonspecific symptoms such as Raynaud’s phenomenon, rarely reported in association with cardiac tumors. It also highlights the importance of considering cardiac masses in the differential diagnosis of pediatric stroke. Prompt surgical management is crucial to prevent fatal embolic events, while long-term follow-up remains essential given the risk of recurrence.

## Introduction

Raynaud’s syndrome is characterized by a triphasic color change - pallor (ischemic phase), cyanosis (deoxygenation phase), and erythema (reperfusion phase) - of the extremities, typically triggered by cold exposure or emotional stress [1]. It is considered rare in childhood, and when present, it is often associated with an underlying cause, often a connective tissue disorder [2].

Primary cardiac tumors are exceptionally rare in children, with an incidence around 0.03%-0.1% [3]. Most are benign, with myxomas representing only a small fraction, typically occurring in older children or adolescents [4]. Atrial myxomas can manifest through a range of nonspecific symptoms, including valvular obstruction, systemic emboli, or constitutional signs, often leading to delayed or missed diagnoses [5]. In fact, we report here the case of a seven-year-old child who had been followed for three years for Raynaud’s syndrome, which was ultimately found to be the initial manifestation of an underlying left atrial myxoma, revealed in the context of an acute ischemic stroke.

## Case presentation

Data for this case were obtained from the patient’s medical records and clinical evaluation.

**Figure 1:**
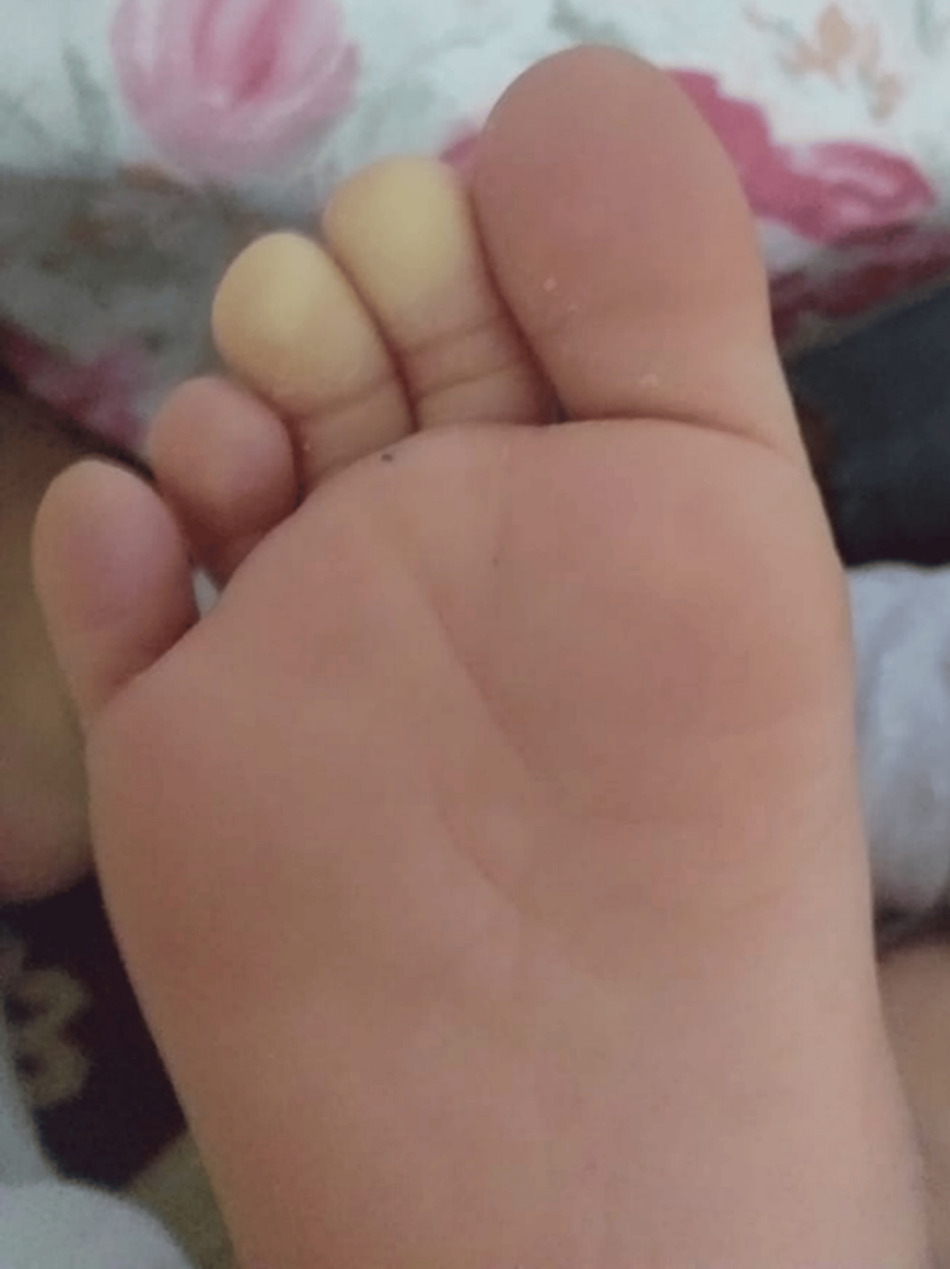
Clinical presentation of Raynaud’s phenomenon in our patient, showing pallor and cyanosis of the toes

A previously healthy seven-year-old boy had been followed since the age of four for episodes of toe discoloration consistent with Raynaud’s phenomenon. These episodes were triggered by cold exposure or walking and characterized by transient pallor and pain of the toes (Figure 1), which significantly limited his physical activity compared to his peers. He also reported exertional dyspnea, which may have been underestimated due to his reduced level of activity. Initial immunological workup and nailfold capillaroscopy were normal, and no underlying systemic disease was identified (Table 1).

**Table 1:** Laboratory Findings in Our Patient Laboratory findings, including initial immunological tests and follow-up blood results after the ischemic stroke.

Test	Result	Reference Range
Antinuclear antibodies (ANA)	0.3	< 0.8
Rheumatoid factor	9.40 IU/ml	< 14
Sodium	137 mmol/L	135–145 mmol/L
Potassium	4.1 mmol/L	3.5–5.1 mmol/L
C-reactive protein (CRP)	2.6 mg/L	0–5 mg/L
Prothrombin time (PT)	90%	70–100%
Hemoglobin	12.10 g/dl	12.9–15.9 g/dl
Platelets	392,000/µl	150,000–400,000/µl
White blood cells (WBC)	8,180/µl	4,000–10,000/µl

Three years later, while Raynaud's phenomenon persisted, the patient acutely presented with right-sided upper limb weakness and facial asymmetry. Neurological examination revealed a right brachial monoparesis and right facial paralysis (NIH Stroke Scale (NIHSS) score: 4). Brain magnetic resonance imaging (MRI) demonstrated a left frontoparietal cortical ischemic stroke. Electrocardiogram and routine blood tests, including coagulation profile and inflammatory markers, were unremarkable.

**Figure 2:**
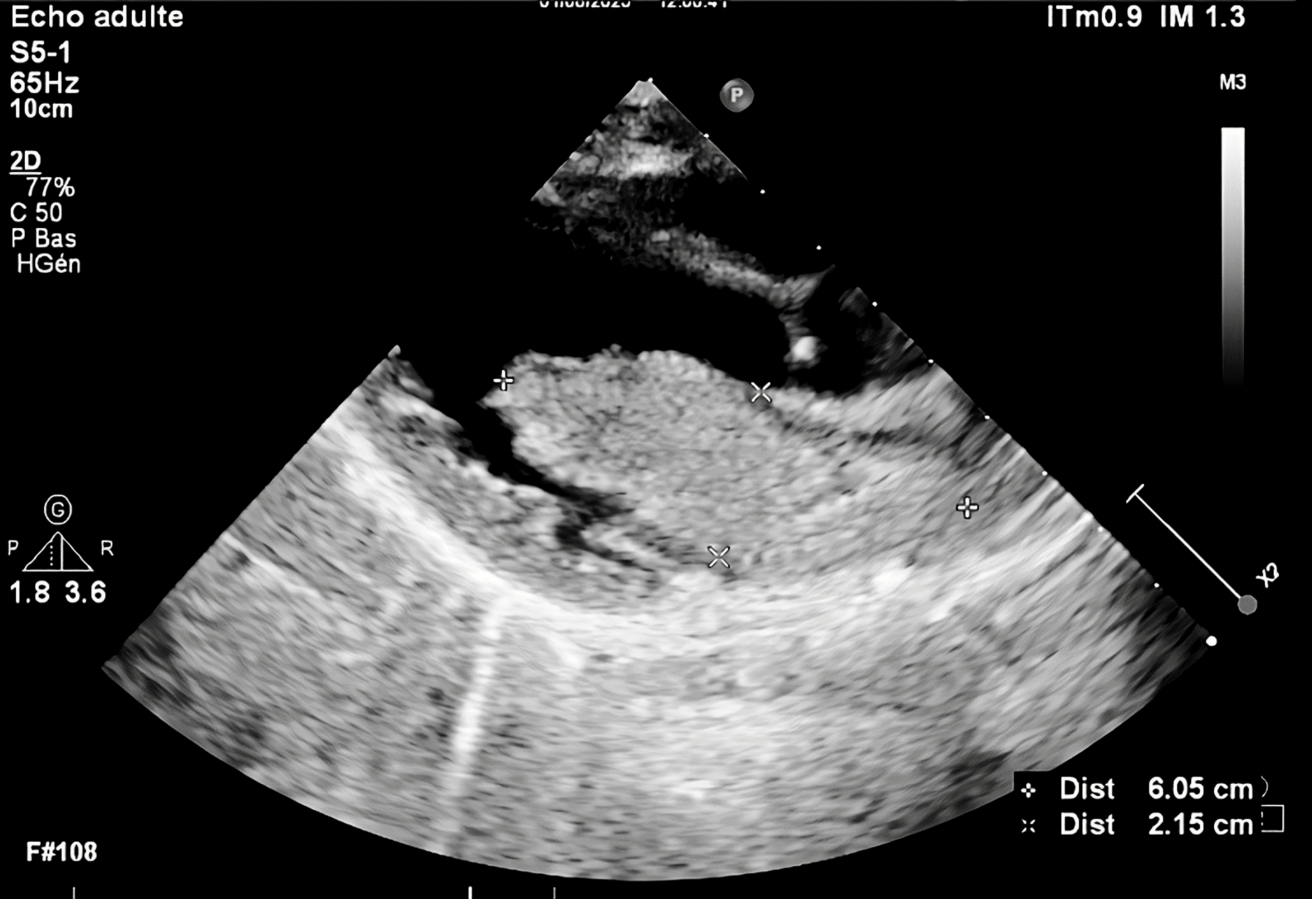
Transthoracic echocardiography showing a large, homogeneous, mobile mass occupying the left atrium, consistent with atrial myxoma

A transthoracic echocardiogram revealed a large, homogeneous, mobile mass completely occupying the left atrium, measuring 60.5 x 21.5 mm, adherent to the interatrial septum, and prolapsing into the left ventricle during diastole, with a mean transmitral gradient of 8 mmHg (Video 1, Figure 2). The echocardiographic features were highly suggestive of a left atrial myxoma [6].

**Video 1:** Apical four-chamber view showing the left atrial myxoma prolabing into the left ventricule

Given the highly emboligenic nature of the tumor and the risk of further potentially fatal cerebral events, an emergency surgical resection was undertaken.

**Figure 3:**
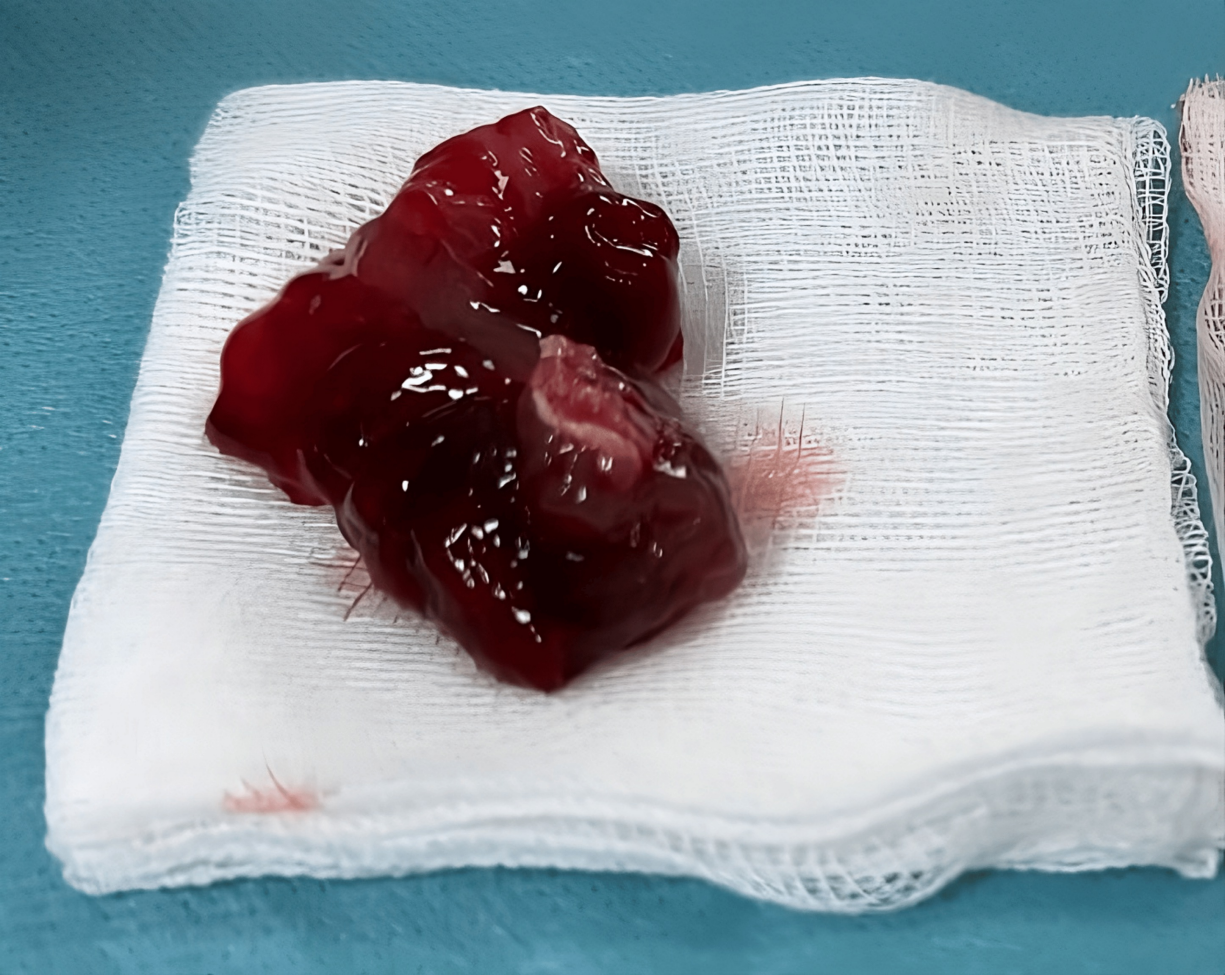
Macroscopic aspect of the left atrial myxoma after surgical resection

It was performed via a median sternotomy under cardiopulmonary bypass with aortic and bicaval cannulation. Following cardioplegic arrest, the right atrium was opened longitudinally to access the interatrial septum, which was incised beginning at the fossa ovalis. This approach exposed a large, fragile, gelatinous, lobulated tumor within the left atrium. The tumor, attached by a pedicle on the interatrial septum, was carefully mobilized and completely excised en bloc, including a full-thickness section of the interatrial septum at its site of attachment (Figure 3).

The mitral valve was examined and found to be intact. Closure of the septal and atrial incisions was performed with running sutures, and the patient was successfully weaned from cardiopulmonary bypass without any drugs and no complication occurred.

The patient was extubated in the intensive care unit after he woke up and postoperative echocardiography didn’t show any residual intra-atrial mass nor mitral regurgitation with a preserved left ventricle. The ECG was normal and the postoperative course in the cardiac intensive care unit was uneventful. The patient was discharged on postoperative day three in good clinical condition. Neurological exam showed a residual hemiparesis.

Macroscopic examination of the excised mass revealed a pedunculated left atrial myxoma measuring 6x4 cm in diameter. The tumor appeared gelatinous, multilobulated, and rounded, with a reddish-brown coloration. Histopathological analysis confirmed the diagnosis, showing an abundant myxoid matrix with numerous isolated stellate-shaped cells scattered throughout the ground substance.

Almost three months postoperatively, the patient showed marked improvement, with resolution of exertional dyspnea and a significant reduction in Raynaud’s episodes, which had previously occurred multiple times daily and are now exceptional.

## Discussion

Cardiac myxomas are the most common primary cardiac tumors in adults, yet they remain exceedingly rare in children, particularly under the age of 12 [4-7]. Reported incidence ranges from 0.03% to 0.1%. Fewer than 20 pediatric cases of left atrial myxoma have been reported in the literature to date [8]. These tumors most frequently arise from the interatrial septum near the fossa ovalis as found in our patient [7].

The clinical presentation of atrial myxomas can be highly variable, often summarized by the classic "Goodwin triad": embolic phenomena, intracardiac obstruction, and constitutional symptoms (e.g., fever, weight loss, or arthralgias) [9].

In children, embolic events are rarer and often unexpected. Our patient came with a left hemispheric ischemic stroke, presenting as right facial and upper limb paralysis.

Interestingly, our patient had a three-year history of Raynaud’s phenomenon with no identifiable autoimmune disease. While extremely rare, Raynaud’s has been described in association with cardiac myxoma, possibly through mechanisms such as microembolization or tumor-related cytokine production (e.g., interleukins) [10]. Although causality cannot be definitively established, the resolution or improvement of Raynaud’s after tumor excision - reported in some cases - suggests a possible link [10].

Diagnostic imaging plays a critical role. Echocardiography, particularly two- and three-dimensional color Doppler, remains the first-line modality to identify intracardiac masses [11,12]. In our case, it revealed a large, mobile, homogeneous mass filling the left atrium and prolapsing into the left ventricle during diastole - findings highly suggestive of a myxoma. The diagnosis was confirmed intraoperatively and by histopathological examination.

Surgical resection is the treatment of choice and should be performed promptly due to the high embolic risk, which may lead to severe complications or even sudden death [11,13]. In our case, surgery was performed urgently with complete tumor excision, including a portion of the atrial septum. The postoperative course was uneventful, and neurological recovery was almost complete.

Long-term follow-up is essential given the 5-7% recurrence risk, particularly in younger patients. Although recurrence is often due to incomplete excision, it can also indicate genetic syndromes such as Carney complex, characterized by multiple myxomas, skin pigmentation, and endocrine abnormalities [14]. At nearly three months post-surgery, the patient showed marked improvement, with a significant reduction in the frequency of Raynaud’s episodes and notable relief of exertional dyspnea.

This case highlights the importance of including cardiac tumors in the differential diagnosis of Raynaud’s phenomenon in children and pediatric ischemic stroke, especially when conventional causes such as arteriovenous malformations or thrombophilia are excluded. Early recognition and surgical management of such tumors can prevent irreversible neurologic damage and reduce mortality.

## Conclusions

This case illustrates the rarity of atrial myxoma in children and its unusual presentation as Raynaud’s phenomenon. The subsequent ischemic stroke revealed the underlying tumor, underscoring the importance of considering cardiac causes in atypical pediatric presentations. Early echocardiographic diagnosis and prompt surgical resection are essential to prevent severe embolic complications, while long-term follow-up is required given the risk of recurrence.
